# Machine learning screening of risk factors for diabetic microvascular complications and construction of a gradient boosting decision tree predictive model

**DOI:** 10.3389/fendo.2026.1784699

**Published:** 2026-04-21

**Authors:** Min Xiao, Yuhao Fu, Yan Li, Qian Liu, Xianyi Qiao, Hongjin Zhang, Xingxing Zhu, Jiajia Wang

**Affiliations:** 1 Department of Laboratory Medicine, Sichuan Provincial People’s Hospital Chuandong Hospital and Dazhou First People’s Hospital, Dazhou, China; 2 College of Medical Technology, Chengdu University of Traditional Chinese Medicine, Chengdu, China; 3 Department of Laboratory Medicine, Sichuan Provincial People’s Hospital, University of Electronic Science and Technology of China, Chengdu, China

**Keywords:** diabetic microvascular complications, gradient boosting decision tree, machine learning, predictive model, risk factors

## Abstract

**Objective:**

To develop a machine learning-based classification model to aid in the early diagnosis of diabetic microvascular complications.

**Methods:**

This study analyzed clinical and laboratory data from 1,498 patients, categorized into two groups: diabetes alone and diabetes with microvascular complications. Independent risk factors for complications were identified through intergroup comparison, collinearity analysis, and logistic regression. Nine machine learning models were subsequently developed and compared. A comprehensive evaluation of the binary classification performance of the Gradient Boosting Decision Tree (GBDT) model was performed.

**Results:**

Urea, fibrinogen (FIB), prothrombin time (PT), D-dimer (DD), creatine kinase MB isoenzyme (CKMB), lipoprotein(a) (Lpa), activated partial thromboplastin time (APTT), triglycerides (TG), and cholinesterase (CHE) were identified as independent risk factors for diabetic microvascular complications. Among the nine predictive models constructed, the GBDT model demonstrated superior performance across multiple metrics, including the area under the receiver operating characteristic curve (AUC) and sensitivity, indicating strong generalization ability on the validation set. Further evaluation confirmed its consistent and robust predictive performance across training, validation, and test datasets. Calibration curve analysis showed good agreement between predicted probabilities and actual outcomes. Decision curve analysis demonstrated the model's clinical utility, and the Kolmogorov-Smirnov (KS) curve indicated excellent discriminatory power.

**Conclusion:**

The GBDT model, constructed based on the identified risk factors, exhibits outstanding predictive performance and promising application potential. It provides important theoretical support and a practical tool for the early identification and targeted intervention of diabetic microvascular complications.

## 1 Introduction

Diabetes mellitus (DM) is a chronic metabolic disorder characterized by hyperglycemia, resulting from the interplay of genetic and environmental factors (1). In China, type 2 diabetes mellitus (T2DM) constitutes approximately 90% of all diabetes cases, with a rising prevalence annually (2). Diabetic retinopathy (DR), diabetic nephropathy (DN), and diabetic peripheral neuropathy (DPN) are among the most common microvascular complications in T2DM patients. These complications represent major causes of blindness, end-stage renal disease, and non-traumatic amputations, significantly impairing patients’ quality of life and survival (3). Consequently, early diagnosis and intervention are critically important for delaying or preventing disease progression and reducing associated disability and mortality.

The risk of microvascular complications increases substantially with the duration of T2DM (3). As a chronic and progressive condition, early-stage diabetic microvascular disease is often reversible, underscoring the importance of timely detection. Current diagnostic methods for DR rely on specialized ophthalmological assessments, such as fundus photography and fluorescein angiography. However, these techniques are not feasible for patients with vitreous hemorrhage, cataracts, or contrast allergies. Moreover, the absence of noticeable symptoms in early DR often leads to delayed diagnosis, limiting the effectiveness of screening programs (4). The diagnosis of DN traditionally relies on renal biopsy, an invasive procedure with restricted clinical utility (5). Alternatively, urinary microalbumin and estimated glomerular filtration rate (eGFR) are commonly used, though both have limitations: urinary microalbumin levels can be influenced by factors such as infection and exercise, while eGFR, derived from serum creatinine, age, and sex, may remain within the normal range in early DN due to renal functional reserve (6). Thus, there is an urgent need to identify sensitive and specific biomarkers. The diagnosis of DPN requires a combination of symptom assessment, physical examination, and ancillary tests. Neurophysiological testing remains an objective standard but lacks sensitivity for early or small-fiber neuropathy and is somewhat invasive, hindering early detection.

Several biomarkers have shown promise for the early detection of diabetic microvascular complications, including dyslipidemia, advanced glycation end products (AGEs), growth factors, and cytokines. These markers are cost-effective, standardized, and suitable for large-scale screening and longitudinal monitoring in primary care settings (7). However, most studies focus on single or a limited number of markers, lacking integrated tools for early risk stratification (8). Recent advances in artificial intelligence and machine learning enable the development of multivariate prediction models. Nonetheless, research systematically integrating multidimensional clinical and laboratory indicators for early warning remains limited.

The main contributions of this study are: For the first time, multidimensional laboratory indicators including coagulation function, cardiac enzyme profiles, lipid profiles, and renal function indicators were integrated with machine learning algorithms to construct an early prediction model for diabetic microvascular complications; through feature importance ranking, key indicators with the greatest contribution to complication prediction were identified, providing an interpretable decision-making basis for clinical practice; nine mainstream machine learning algorithms were systematically compared, confirming the superiority of the GBDT model for this prediction task. This integrated analysis strategy not only provides a new technical approach for early warning of diabetic microvascular complications but also lays a theoretical foundation for future biomarker research and development of personalized intervention strategies (7).

## 2 Materials and methods

### 2.1 Study population and data collection

This study included 1,702 diabetic patients admitted to Sichuan Provincial People’s Hospital between January 1, 2019, and December 31, 2023. Basic demographic and clinical information was collected upon admission, and fasting venous blood samples were obtained for complete blood count, glycated hemoglobin (HbA1c), coagulation function, and biochemical testing. After excluding patients with missing data rates exceeding 30%, 1,498 patients were retained for final analysis, including 424 with diabetes alone and 1,074 with diabetes and microvascular complications. Exclusion criteria were: non-diabetic microvascular disease (e.g., hypertensive nephrosclerosis, immune-mediated nephropathy); history of major cardiovascular or cerebrovascular events; Severe hepatic or renal impairment (eGFR < 30 mL/min/1.73m²); concurrent malignancy or autoimmune disease; Pregnancy or lactation; Critical data missing (e.g., fundus examination or nerve conduction studies).

### 2.2 Data preprocessing and feature selection

#### 2.2.1 Data preprocessing

This study implemented a rigorous multi-step quality control process. First, samples with a missing rate exceeding 30% were excluded. Second, outlier detection was performed, where values exceeding mean ± 3 standard deviations were considered potential outliers; after verification against original medical records, confirmed data entry errors were corrected, while biologically plausible but abnormal values were retained. Third, consistency checks were conducted to resolve logical inconsistencies (such as conflicting diagnoses or laboratory values inconsistent with clinical presentation) by cross-referencing electronic health records. Fourth, only patients with complete records for key diagnostic criteria (fundus examination for DR, urinary albumin-to-creatinine ratio for DN, and nerve conduction studies for DPN) were included. Ultimately, out of 1,702 initial patients, 1,498 were included in the analysis.

#### 2.2.2 Missing value handling

After excluding samples with a missing rate >30%, the remaining missing values were imputed using the random forest algorithm. With complete variables as predictors and variables containing missing values as target variables, a random forest model was constructed based on 100 decision trees, and imputation accuracy was assessed through out-of-bag error. All imputed values for continuous variables were normalized to ensure scale consistency.

#### 2.2.3 Dataset splitting

Using stratified random sampling, the 1,498 samples were divided into a training set (n=1,273) and a test set (n=225) at an 85:15 ratio, ensuring that the proportion of diabetic microvascular complications remained consistent between the two groups. The training set was further subjected to 10-fold cross-validation for model training and hyperparameter tuning. The training set was further subjected to 10-fold cross-validation for model training and hyperparameter tuning. All analyses were performed using R (v4.2.2) and SPSS (v26.0). Continuous variables were summarized as mean ± standard deviation (SD) if normally distributed, or median with interquartile range (IQR) otherwise, and compared using t-tests or Mann-Whitney U tests. Categorical variables were described as frequencies and percentages and compared using chi-square or Fisher’s exact tests. Multicollinearity was assessed using the variance inflation factor (VIF), with VIF > 5 indicating significant collinearity. Binary logistic regression was used to select statistically significant predictors for subsequent modeling.

### 2.3 Model development and evaluation

Nine machine learning algorithms were compared: XGBoost, LightGBM, Random Forest, AdaBoost, Decision Tree, Gradient Boosting (GBDT), Gaussian Naïve Bayes, Complement Naïve Bayes, and Multilayer Perceptron (MLP) Classifier. Model performance was evaluated primarily using the area under the ROC curve (AUC), supplemented by accuracy, sensitivity, specificity, positive predictive value (PPV), negative predictive value (NPV), F1-score, and Kappa coefficient. The best-performing model was selected for further validation of its classification performance. Hyperparameter Optimization Strategy: This study employed grid search combined with 10-fold cross-validation for hyperparameter tuning of all nine machine learning models. The specific process was as follows: (1) Search space definition: For each model’s key hyperparameters, reasonable search ranges and step sizes were determined based on literature review and preliminary experiments. (2) Grid search implementation: Each hyperparameter combination was evaluated on the training set using 10-fold cross-validation, with the mean AUC on the validation set as the optimization objective. The hyperparameter combination that maximized the AUC was selected as the optimal parameter set for that model; (3) Early stopping mechanism: For models supporting incremental learning (XGBoost, LightGBM, GBDT), early stopping was additionally applied on top of grid search, terminating training when validation set performance did not improve for 10 consecutive rounds, to further prevent overfitting.

Taking the GBDT model as an example, its hyperparameter optimization range included: n_estimators searched in the range [50, 200] with a step size of 10, max_depth searched in the range [3, 10] with a step size of 1, min_samples_split searched in the range [2, 10] with a step size of 1, min_samples_leaf searched in the range [1, 5] with a step size of 1, learning_rate searched in the range [0.01, 0.3] with a step size of 0.05, and subsample searched in the range [0.6, 1.0] with a step size of 0.1. The optimal combination was selected from 64,800 parameter combinations through 10-fold cross-validation (see **Supplementary Table 1** for details).

## 3 Results

### 3.1 Baseline characteristics

A total of 1,702 diabetic patients were initially enrolled. After quality control, 1,498 patients were included in the analysis, comprising 424 with diabetes alone and 1,074 with microvascular complications. Comparative analysis revealed statistically significant differences (*P* < 0.05) between the two groups across 33 clinical and laboratory variables (**Table 1**).

**Table 1:** Comparison of baseline characteristics between diabetes and diabetes with microvascular complications.

Variables	Diabetes (n=424)	Diabetes with microvascular complications (n=1074)	*P*-value
Patient characteristics			
Age	57.500 (45, 68)	65.000 (54, 75)	<0.001
Sex			0.253
Male	154 (36.321%)	355 (33.054%)	
Female	270 (63.679%)	719 (66.946%)	
Laboratory indices			
White blood cell (×10^9^/L)	6.28 (5.25, 7.40)	6.31 (5.17, 7.62)	0.453
Neutrophil (×10^9^/L)	3.98 (3.06, 4.77)	4.21 (3.31, 5.30)	<0.001
Lymphocyte (×10^9^/L)	1.66 (1.36, 2.11)	1.40 (1.07, 1.80)	<0.001
Platelet (×10^9^/L)	186.76 (147.75, 224.25)	185.00 (142.00, 225.00)	0.542
Hemoglobin(g/L)	143.00 (131.00, 153.00)	130.00 (111.00, 145.00)	<0.001
Hs C-reactive protein (pg/ml)	6.05 (3.69, 9.88)	2.22 (0.95, 6.27)	<0.001
Hemoglobin A1c (%)	9.21 (7.50, 11.19)	8.33 (7.15, 10.15)	<0.001
Fibrinogen (g/L)	2.83 (2.34, 3.43)	3.35 (2.83, 4.12)	<0.001
D-dimer (mg/L)	0.28 (0.19, 0.48)	0.32 (0.19, 0.79)	<0.001
Fibrin Degradation Products (μg/ml)	2.40 (1.30, 2.50)	2.50 (2.50, 3.24)	<0.001
Thrombin Time (s)	17.50 (16.90, 18.13)	17.40 (16.70, 18.00)	0.009
Prothrombin Time (s)	11.00 (10.50, 11.50)	11.50 (10.80, 12.50)	<0.001
Activated partial thromboplastintime (s)	25.62 (23.70, 27.40)	26.80 (25.30, 28.50)	<0.001
Antithrombin III (%)	138.30 (124.20, 150.89)	147.17 (143.51, 168.99)	<0.001
Total bilirubin (umol/L)	4.15 (2.73, 7.20)	4.90 (2.90, 7.90)	<0.001
Direct bilirubin (umol/L)	3.90 (2.90, 4.90)	3.70 (2.80, 4.90)	0.185
Indirect bilirubin (umol/L)	7.20 (5.00, 10.50)	6.30 (4.00, 9.25)	<0.001
Total Protein (g/L)	66.95 (62.20, 71.43)	67.90 (62.70, 72.60)	0.150
Albumin (g/L)	40.95 (38.10, 44.13)	40.40 (36.10, 44.00)	0.005
Globulin (g/L)	25.70 (22.40, 29.23)	27.25 (23.80, 31.10)	<0.001
Urea nitrogen (mmol/L)	53.10 (4.270, 6.68)	7.44 (5.71, 10.70)	<0.001
Creatinine (umol/L)	64.15 (53.90, 76.00)	84.00 (64.83, 131.100)	<0.001
Uric acia (umol/L)	329.50 (275.00, 396.00)	343.5 (275.25, 419.00)	0.040
Serum calcium (mmol/L)	2.28 (2.20, 2.35)	2.29 (2.18, 2.39)	0.459
Serum Magnesium (mmol/L)	0.83 (0.78, 0.89)	0.85 (0.79, 0.91)	0.006
Serum Phosphorus (mmol/L)	1.13 (1.04, 1.23)	1.12 (0.99, 1.27)	0.308
Glucose (mmol/L)	9.75 (6.92, 14.13)	10.09 (7.16, 15.03)	0.424
Total cholesterol (mmol/L)	4.42 (3.88, 5.14)	4.27 (3.65, 4.80)	<0.001
Triglyceride (mmol/L)	2.16 (1.63, 3.02)	1.55 (1.04, 2.34)	<0.001
High density lipoprotein (mmol/L)	1.08 (0.92, 1.26)	1.05 (0.91, 1.21)	0.060
low density lipoprotein (mmol/L)	2.63 (2.13, 3.24)	2.54 (1.89, 2.87)	<0.001
Apolipoprotein A1 (g/L)	1.24 (1.10, 1.38)	1.23 (1.13, 1.39)	0.31
Apolipoprotein B (g/L)	0.91 (0.76, 1.07)	0.90 (0.74, 1.02)	0.062
Lipoprotein(a) (mg/dL)	143.29 (103.15, 189.30)	36.50 (12.00, 108.50)	<0.001
Cholinesterase (U/L)	8.00 (6.69, 9.50)	7.78 (6.30, 9.20)	<0.001
Cystatin C (mg/L)	0.92 (0.79, 1.11)	1.20 (0.92, 1.79)	<0.001
Procalcitonin (ng/L)	0.17 (0.11, 0.27)	0.14 (0.09, 0.25)	<0.001
High-Sensitivity Cardiac Troponin T (ng/L)	28.32 (20.83, 42.05)	17.69 (9.15, 30.61)	<0.001
Ferritin (ng/L)	276.35 (172.04, 394.50)	286.89 (187.70, 398.71)	0.215
Brain Natriuretic Peptide (pg/L)	140.82 (91.17, 208.40)	77.46 (32.15, 165.51)	<0.001
Myoglobin (ng/L)	87.23 (58.74, 117.83)	50.88 (32.68, 81.40)	<0.001
Creatine Kinase Myocardial Band (U/L)	16.80 (14.84, 22.74)	14.90 (12.00, 17.17)	<0.001

Continuous variables are presented as median (25th percentile, 75th percentile); categorical variables are presented as frequency (percentage). A P-value <0.05 indicates statistical significance.

### 3.2 Risk factor selection

Multicollinearity diagnosis indicated that IBIL, TBIL, Cystatin C (CysC), Creatinine (Crea), and eGFR had VIF>5, leading to their exclusion. Univariate and multivariate logistic regression analyzes performed on the remaining 28 variables identified nine independent risk factors: Urea, FIB, PT, DD, CKMB, Lpa, APTT, TG, and CHE (**Table 2**). Based on the nine independent risk factors identified through the above multivariate logistic regression analysis, we further constructed nine machine learning models, aiming to compare the performance of different algorithms in predicting diabetic microvascular complications and to select the model most suitable for clinical translation.

**Table 2:** Univariate logistic regression and multivariate logistic regression.

Parameter	Univariable		Multivariable	
	OR (95% CI)	*p*-value	OR (95% CI)	*p*-value
TC	0.88 (0.79, 0.98)	0.02	1.09 (0.81, 1.47)	0.555
LDLC	0.81 (0.71, 0.93)	0.003	0.79 (0.55, 1.13)	0.201
Urea	1.36 (1.28, 1.45)	<0.001	1.29 (1.20, 1.39)	<0.001
Hb	0.98 (0.97, 0.98)	<0.001	1.00 (0.99, 1.01)	0.656
Age	1.04 (1.03, 1.05)	<0.001	1.01 (1.00, 1.02)	0.097
Alb	0.96 (0.94, 0.98)	<0.001	1.04 (1.00, 1.07)	0.052
FIB	1.68 (1.45, 1.95)	<0.001	1.27 (1.06, 1.53)	0.01
UA	1.00 (1.00, 1.00)	0.106		
N	1.06 (0.99, 1.14)	0.093		
HbA1c	0.90 (0.85, 0.95)	<0.001	1.04 (0.97, 1.12)	0.274
FDP	1.38 (1.23, 1.55)	<0.001	1.02 (0.95, 1.10)	0.608
Mg	2.34 (0.74, 7.42)	0.148		
L	0.49 (0.40, 0.61)	<0.001	0.83 (0.62, 1.10)	0.198
Glb	1.05 (1.02, 1.07)	<0.001	1.03 (0.99, 1.06)	0.107
PCT	1.17 (0.91, 1.50)	0.21		
ATIII	1.00 (1.00, 1.01)	0.004	1.00 (0.99, 1.00)	0.282
PT	1.79 (1.56, 2.05)	<0.001	1.62 (1.37, 1.92)	<0.001
MYO	1.00 (1.00, 1.00)	0.006	1.00 (1.00, 1.00)	0.266
DD	1.48 (1.20, 1.83)	<0.001	1.38 (1.12, 1.71)	0.003
CKMB	0.95 (0.93, 0.96)	<0.001	0.98 (0.96, 0.99)	0.007
Lpa	1.00 (1.00, 1.00)	<0.001	1.00 (1.00, 1.00)	<0.001
hscTnT	1.00 (1.00, 1.00)	0.041	1.00 (1.00, 1.00)	0.124
hsCRP	1.00 (0.99, 1.01)	0.746		
BNP	1.00 (1.00, 1.00)	0.392		
APTT	1.18 (1.13, 1.24)	<0.001	1.16 (1.10, 1.22)	<0.001
TG	0.90 (0.86, 0.95)	<0.001	0.91 (0.85, 0.97)	0.007
TT	1.02 (0.98, 1.05)	0.376		
CHE	0.80 (0.76, 0.84)	<0.001	0.81 (0.76, 0.86)	<0.001

### 3.3 Model performance comparison and comprehensive evaluation of the GBDT model

#### 3.3.1 Performance comparison of nine machine learning models

**Figure 1:**
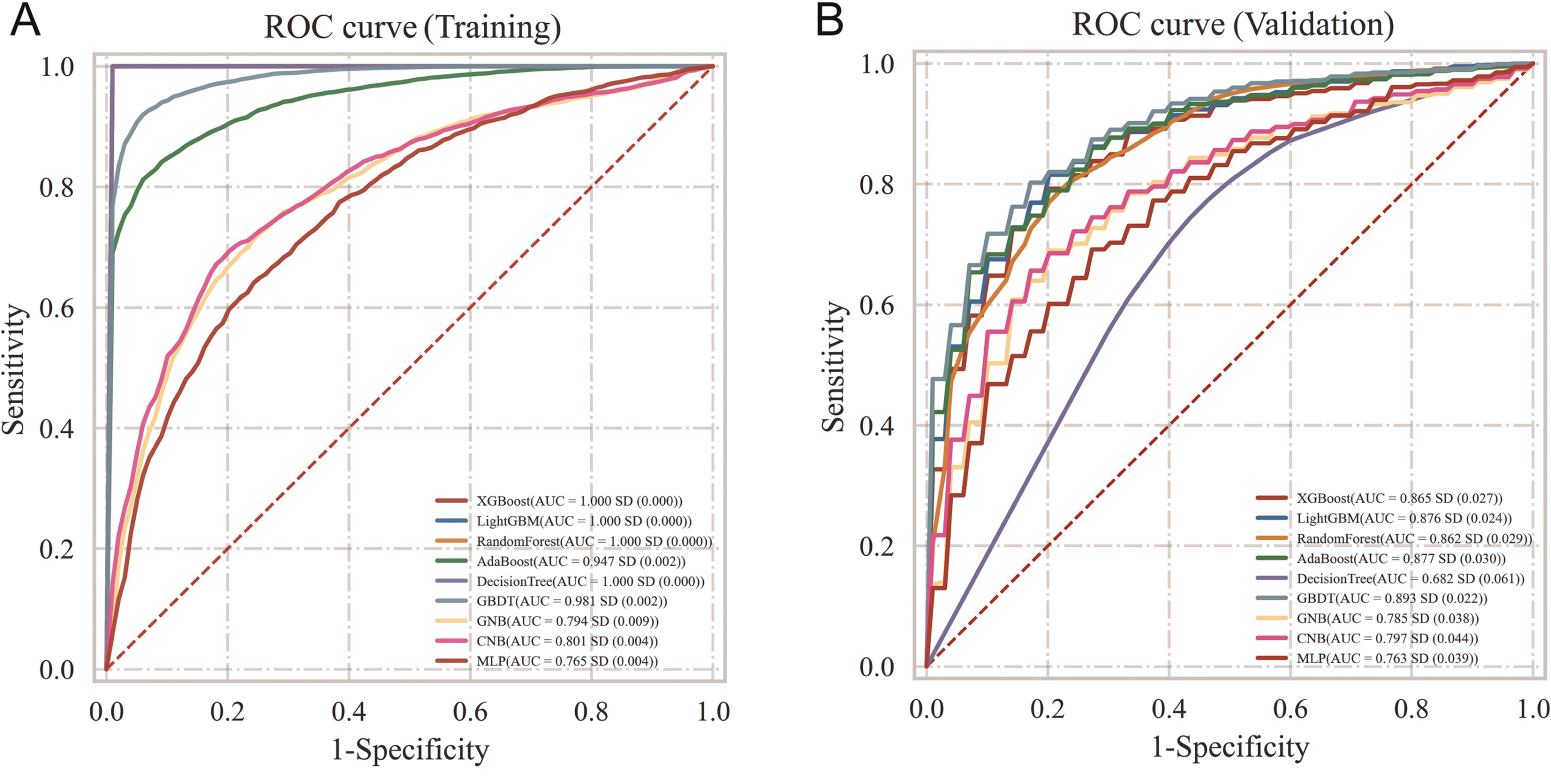
**(A)** Model performance comparison on the training set. **(B)** Model performance comparison on the validation set.

Based on the nine identified independent risk factors, nine machine learning classification models were constructed in this study. As shown in **Table 3**, on the training set, XGBoost, LightGBM, and Random Forest achieved near-perfect AUC values (1.000, 0.999-1.000) (**Figure 1**), indicating the strong fitting capability of these ensemble learning models. However, on the validation set (**Table 4**), model performance diverged: XGBoost’s AUC decreased to 0.865, LightGBM to 0.876, and Random Forest to 0.862, suggesting a certain degree of overfitting in these models.

**Table 3:** Performance comparison of nine machine learning models on the training set.

Classification model	Cutoff (SD)	Accuracy (SD)	Sensitivity (SD)	Specificity (SD)	F1 score (SD)	AUC (95% CI)
XGBoost	0.888 (0.006)	1.000 (0.000)	1.000 (0.000)	1.000 (0.000)	1.000 (0.000)	1.000 (0.000)
LightGBM	0.865 (0.013)	1.000 (0.000)	1.000 (0.000)	1.000 (0.000)	1.000 (0.000)	1.000 (0.000)
RandomForest	0.610 (0.030)	0.999 (0.001)	0.998 (0.001)	1.000 (0.001)	0.999 (0.001)	1.000 (0.000)
AdaBoost	0.508 (0.001)	0.852 (0.011)	0.830 (0.017)	0.931 (0.016)	0.897 (0.008)	0.947 (0.002)
DecisionTree	1.000 (0.000)	1.000 (0.000)	1.000 (0.000)	1.000 (0.000)	1.000 (0.000)	1.000 (0.000)
GBDT	0.729 (0.018)	0.925 (0.007)	0.919 (0.011)	0.946 (0.011)	0.950 (0.005)	0.981 (0.002)
GNB	0.923 (0.192)	0.727 (0.020)	0.717 (0.032)	0.763 (0.029)	0.804 (0.019)	0.794 (0.009)
CNB	0.501 (0.000)	0.712 (0.009)	0.683 (0.016)	0.817 (0.017)	0.787 (0.009)	0.801 (0.004)
MLP	0.776 (0.003)	0.671 (0.028)	0.647 (0.051)	0.756 (0.054)	0.754 (0.029)	0.765 (0.004)

**Table 4:** Performance comparison of the nine machine learning models on the validation set.

Classification model	Cutoff (SD)	Accuracy (SD)	Sensitivity (SD)	Specificity (SD)	F1 score (SD)	AUC (95% CI)
XGBoost	0.888 (0.006)	0.798 (0.038)	0.817 (0.036)	0.730 (0.059)	0.863 (0.028)	0.865 (0.027)
LightGBM	0.865 (0.013)	0.821 (0.027)	0.840 (0.029)	0.753 (0.060)	0.880 (0.019)	0.876 (0.024)
RandomForest	0.610 (0.030)	0.834 (0.017)	0.905 (0.028)	0.580 (0.095)	0.895 (0.011)	0.862 (0.029)
AdaBoost	0.508 (0.001)	0.789 (0.034)	0.795 (0.045)	0.767 (0.070)	0.854 (0.027)	0.877 (0.030)
DecisionTree	1.000 (0.000)	0.781 (0.029)	0.858 (0.028)	0.507 (0.127)	0.860 (0.018)	0.682 (0.061)
GBDT	0.729 (0.018)	0.825 (0.024)	0.851 (0.022)	0.730 (0.060)	0.883 (0.016)	0.893 (0.022)
GNB	0.923 (0.192)	0.716 (0.029)	0.708 (0.046)	0.747 (0.067)	0.795 (0.028)	0.785 (0.038)
CNB	0.501 (0.000)	0.702 (0.035)	0.675 (0.042)	0.797 (0.057)	0.779 (0.031)	0.797 (0.044)
MLP	0.776 (0.003)	0.658 (0.051)	0.641 (0.080)	0.720 (0.087)	0.743 (0.051)	0.763 (0.039)

In contrast, the GBDT model achieved a training set AUC of 0.981 (95% CI: 0.979-0.983) and a validation set AUC of 0.893 (95% CI: 0.871-0.915), showing the smallest performance decline and demonstrating the best generalization capability. On other evaluation metrics in the validation set, the GBDT model also showed balanced performance: accuracy 0.825, sensitivity 0.851, specificity 0.730, F1 score 0.883. Naive Bayes class models (GNB, CNB) and MLP scored lower than ensemble learning models on all metrics, suggesting that for the nonlinear, high-dimensional data characteristics of this study, ensemble learning methods have significant advantages.

#### 3.3.2 Validation of the GBDT model on an independent test set

**Figure 2:**
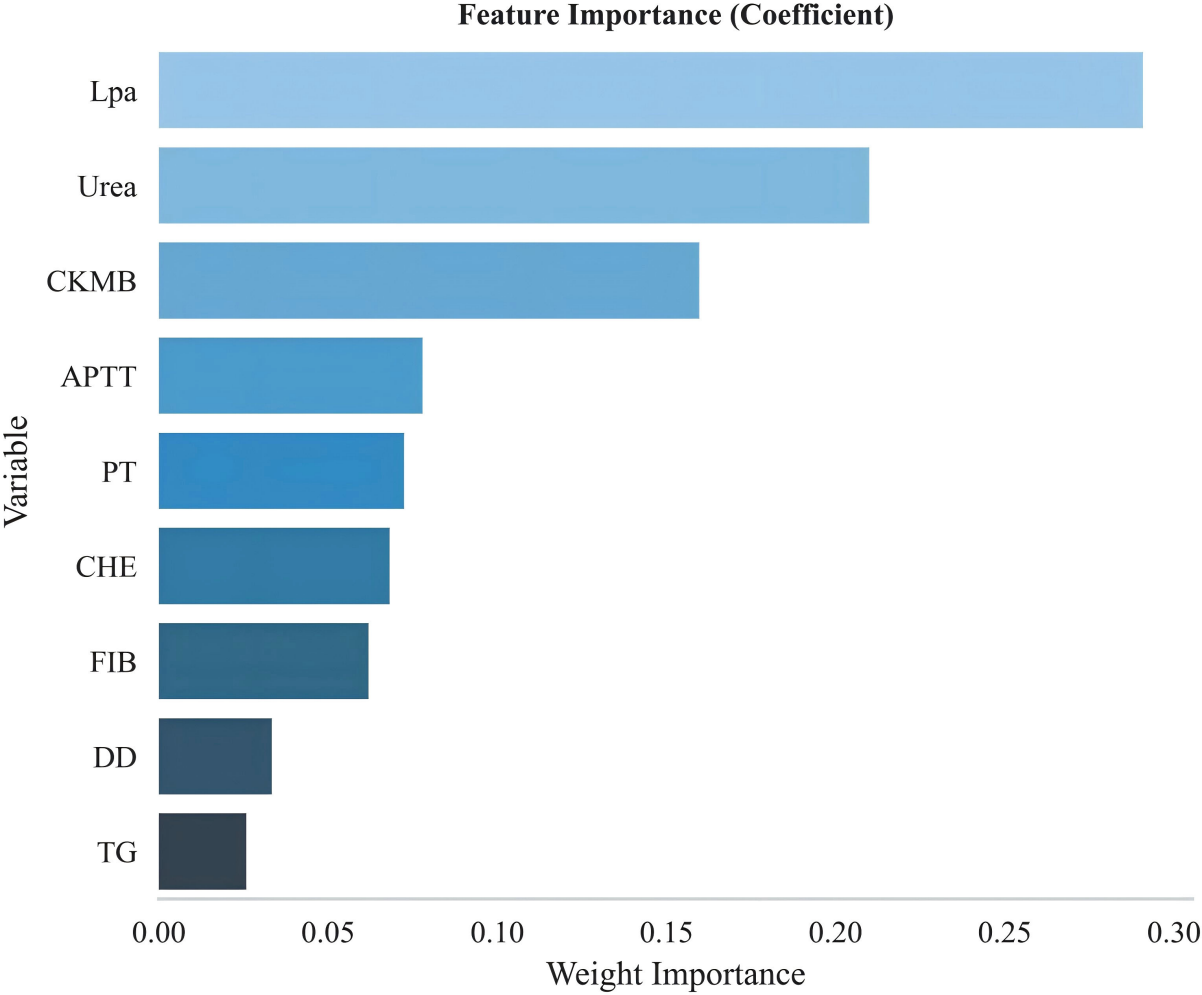
Variable importance ranking.

**Figure 3:**
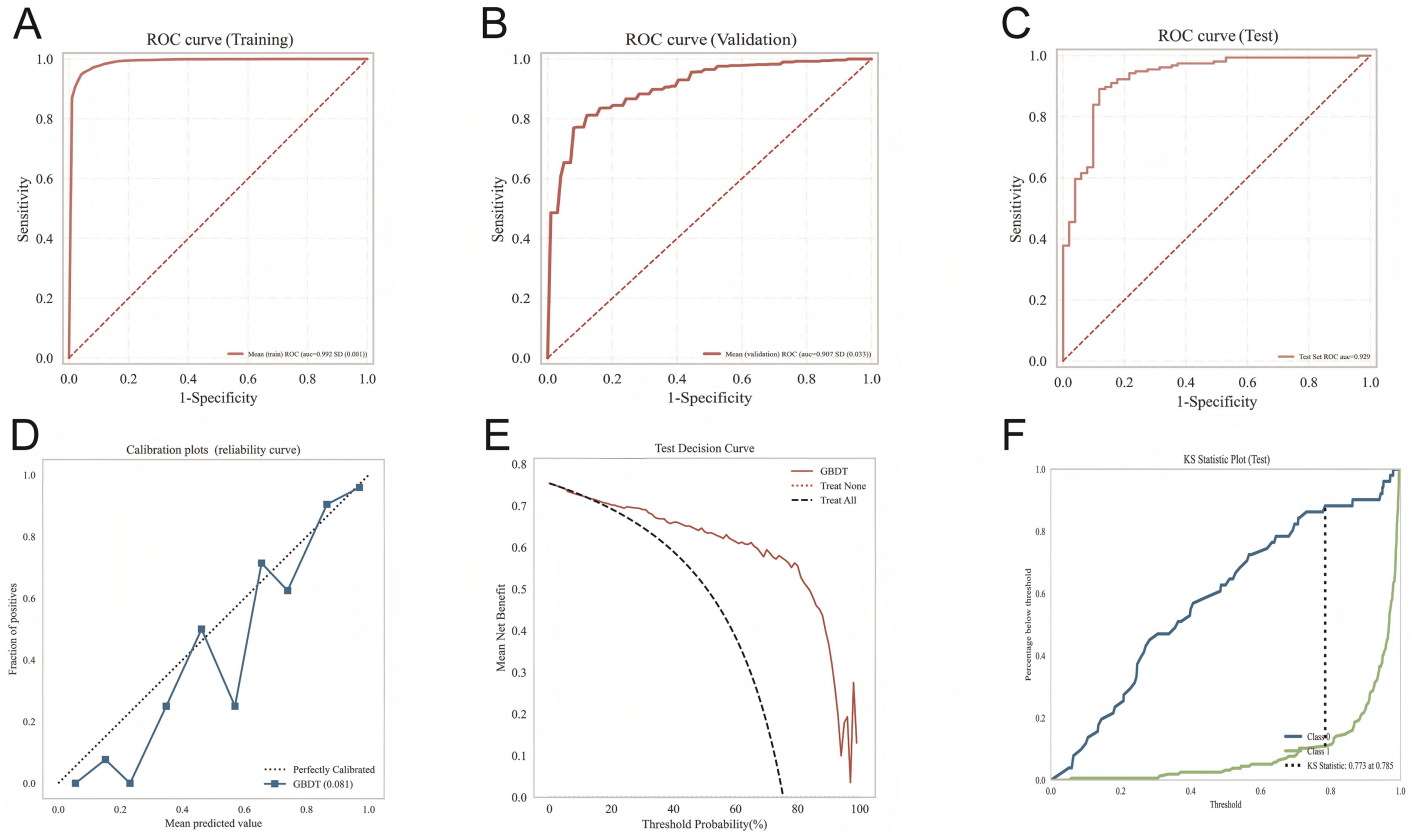
**(A)** GBDT model training set ROC curve. **(B)** GBDT model validation set ROC curve. **(C)** GBDT model test set ROC curve. **(D)** GBDT model calibration curve. **(E)** GBDT model clinical decision curve. **(F)** GBDT model KS curve.

The importance ranking of the nine variables in the GBDT model is Lpa, Urea, CKMB, APTT, PT, CHE, FIB, DD, TG (**Figure 2**). The variable importance ranking not only has statistical significance but also contains important pathophysiological insights. Lipoprotein a (Lpa) ranked first—a finding worthy of in-depth exploration. Lpa is a genetically determined lipoprotein particle, and recent studies have confirmed its dual role in promoting atherosclerosis and thrombosis (9, 10). Lpa being identified as the most important predictor in this study suggests that Lpa may be involved not only in macrovascular disease but also plays a key role in microvascular injury—a hypothesis worthy of validation in future basic research. Urea ranked second, reflecting the central role of renal impairment in microvascular complications, consistent with the pathophysiological process of diabetic nephropathy (11). Notably, coagulation-related indicators (APTT, PT, FIB, DD) collectively occupy four positions, with cumulative importance exceeding 30%, strongly suggesting the important role of hypercoagulable states and microthrombus formation in the pathogenesis of diabetic microvascular complications (12–14). This finding has direct clinical implications: for diabetic patients, in addition to routine monitoring of blood glucose and renal function, regular assessment of coagulation function may help in early identification of microvascular complication risk. To further validate the generalization capability of the GBDT model, we evaluated its performance on an independent test set of 225 cases. Results showed (**Figure 3C**) that the model achieved an AUC of 0.9289, accuracy of 0.8841, sensitivity of 0.8972, and specificity of 0.8545, indicating that the model maintains stable predictive performance on unseen data. **Figures 3A–C** display the ROC curves of the GBDT model on the training, validation, and test sets. The three curves have similar shapes with AUC values all exceeding 0.90, confirming the model’s good stability and generalization capability. Notably, the test set AUC (0.9289) is slightly higher than the validation set AUC (0.907). This seemingly counterintuitive phenomenon may be attributed to the similarity in distribution between the test set and training set, as well as random fluctuations in validation set samples during the 10-fold cross-validation process. The calibration curve (**Figure 3D**) is a key indicator for assessing clinical reliability of the model—an ideal model should lie near the 45-degree diagonal line. In this study, the calibration curve closely aligns with the diagonal within the 0.2-0.8 predicted probability range, with no significant difference in the Hosmer-Lemeshow test (P>0.05), indicating that the risk probabilities provided by the model are highly consistent with actual occurrence probabilities. This characteristic is crucial for clinical risk communication and treatment decisions.

#### 3.3.3 Clinical utility analysis

Decision curve analysis (**Figure 3E**) further translates model performance into clinical value: within the 0.2-0.8 threshold range, the net benefit of using the GBDT model to guide intervention decisions is higher than “treat all” or “treat none” strategies. This means that in clinical practice, this model can help physicians more accurately identify high-risk patients and avoid unnecessary interventions for low-risk patients. The maximum distance of 0.733 in the KS curve (**Figure 3F**) indicates that the model can effectively distinguish between high-risk and low-risk populations, providing a quantitative basis for risk stratification. Synthesizing the results from ROC curves, calibration curves, decision curves, and KS curves, the GBDT model demonstrates excellent performance across four dimensions: predictive accuracy, calibration, clinical net benefit, and discriminative ability. Below, we discuss the clinical significance of these findings and study limitations in depth, integrating with existing literature.

## 4 Discussion

This study systematically identified key risk factors and developed a predictive model for diabetic microvascular complications using machine learning. The principal findings include: (1) 33 variables exhibited significant differences between patients with and without complications; (2) 9 independent risk factors were identified; (3) variable importance was quantitatively ranked; (4) the GBDT model outperformed other algorithms; and (5) the model demonstrated robust binary classification performance for early risk prediction.

The identified biomarkers Urea, FIB, PT, DD, CKMB, Lpa, APTT, TG, and CHE—align with known pathophysiological mechanisms. Lpa, which ranked highest in importance, is involved in lipoprotein metabolism and vascular inflammation and has been increasingly linked to microvascular injury (9, 10). Elevated Urea levels reflect renal impairment associated with DN (11). Abnormalities in CKMB may indicate cardiac microvascular involvement (15). Coagulation markers (PT, APTT, FIB, DD) suggest a hypercoagulable state contributing to microvascular thrombosis (12–14). Elevated TG levels are tied to insulin resistance and metabolic dysregulation (16).

Multicollinearity was addressed by excluding highly correlated variables (VIF > 5). Future studies could employ regularization techniques or principal component analysis for further optimization. Among the models tested, GBDT showed balanced performance without evident overfitting, leveraging its ensemble structure to capture complex nonlinear relationships—characteristics that render it suitable for clinical risk prediction (17, 18). Its performance advantages over traditional models, such as logistic regression or single decision trees, have been consistently reported (7, 19). The model’s interpretability, aided by feature importance rankings, enhances its clinical translatability and fosters trust among physicians (20, 21).

In clinical practice, this model may facilitate the early screening of high-risk individuals, enabling timely intervention and improving prognosis. The multi-biomarker approach offers a non-invasive, scalable strategy for assessing the risk of complications.

This study has several limitations. Its single-center design and limited sample size may affect the generalizability of the findings. The lack of long-term follow-up data prevents the assessment of biomarker dynamics and prognostic value. Experimental validation of the identified biomarkers (e.g., Lpa) is lacking, limiting mechanistic insight. Potential batch effects due to limited dataset diversity may influence model stability. Explanation of Hyperparameter Tuning Strategy: In this study, grid search combined with 10-fold cross-validation was employed to optimize the hyperparameters of all models, with the search ranges determined based on literature review and preliminary experiments. The final optimal hyperparameters for each model have been listed in **Supplementary Table 1**. It should be noted that the statement in the original manuscript regarding the “lack of complete documentation” was inappropriately phrased and may have caused misunderstandings; we have deleted this statement in the revised manuscript and clarified as follows: The tuning process in this study was systematic and standardized, with all models evaluated and selected under a unified optimization framework. We recommend that future studies adopt more systematic experimental recording tools (such as MLflow, TensorBoard, etc.) when conducting similar work to automatically document all attempted parameter combinations, thereby further enhancing research transparency and reproducibility.

We encourage other researchers to build upon our findings with more systematically documented approaches. Future multicenter, large-sample prospective studies combined with molecular validation are needed to enhance reliability and clinical utility. Reproducibility Assurance Measures: To ensure the reproducibility of this study, we have taken the following measures: (1) detailed reporting of each model’s hyperparameter search space and optimization process; (2) public release of the complete analysis code, including the specific implementation of hyperparameter tuning; (3) provision of a parameter optimization example using the GBDT model in the Methods section to facilitate readers’ understanding of the tuning process. Although the complete search space documentation has been provided as comprehensively as possible, we still recommend that future studies adopt more systematic experimental recording practices when conducting similar work (such as using tools like MLflow or TensorBoard to automatically record all attempted parameter combinations), to further enhance research transparency and reproducibility.

## 5 Conclusion

This study identified and validated key risk factors for diabetic microvascular complications and constructed a high-performance GBDT prediction model. The model demonstrates excellent discriminative ability, calibration, and clinical utility, providing a practical tool for early risk assessment and personalized management. Despite its limitations, these findings enhance our understanding of the pathogenesis of diabetic microvascular complications and offer a valuable approach for improving patient outcomes.

## Data Availability

The original contributions presented in the study are included in the article/**Supplementary Material**. Further inquiries can be directed to the corresponding author.

## Supplementary material

Supplementary Table 1 Final optimal hyperparameters for nine machine learning models.
