# Supplementary material for: Machine learning screening of risk factors for diabetic microvascular complications and construction of a gradient boosting decision tree predictive model
Source: Front Endocrinol (Lausanne). 2026 Apr 21;17:1784699. doi: 10.3389/fendo.2026.1784699 (PMC13139028; doi:10.3389/fendo.2026.1784699)
Supplement: Supplementary Table 1 — Final optimal hyperparameters for nine machine learning models. [file DataSheet1.docx]

**Supplementary Table S1.** Final Optimal Hyperparameters for Nine Machine Learning Models

| Model | Key Hyperparameters | Final Optimal Values |
| --- | --- | --- |
| XGBoost | n_estimators, max_depth, learning_rate, subsample | 100, 5, 0.1, 0.8 |
| LightGBM | n_estimators, max_depth, num_leaves, learning_rate | 100, 5, 31, 0.1 |
| ndom Forest | n_estimators, max_depth, min_samples_split, min_samples_leaf | 100, 10, 5, 2 |
| GBDT | n_estimators, max_depth, min_samples_split, min_samples_leaf, learning_rate, subsample | 100, 5, 5, 2, 0.1, 0.8 |
| aBoost | n_estimators, learning_rate | 100, 1.0 |
| Decision Tree | max_depth, min_samples_split, min_samples_leaf | 5, 2, 1 |
| GNB | priors | None |
| CNB | alpha | 1.0 |
| MLP | hidden_layer_sizes, activation, solver, max_iter | (100,), 'relu', 'adam', 500 |
